# The course of health-related quality of life in the first 2 years after a diagnosis of head and neck cancer: the role of personal, clinical, psychological, physical, social, lifestyle, disease-related, and biological factors

**DOI:** 10.1007/s00520-023-07918-w

**Published:** 2023-07-11

**Authors:** Irma M. Verdonck-de Leeuw, Laura H.A. Korsten, Annette van Nieuwenhuizen, Rob J. Baatenburg de Jong, Ruud H. Brakenhoff, Laurien M. Buffart, Femke Lamers, Johannes A. Langendijk, C. René Leemans, Jan H. Smit, Mirjam A. Sprangers, Robert P. Takes, Chris H. J. Terhaard, Birgit I. Lissenberg-Witte, Femke Jansen

**Affiliations:** 1 Department Otolaryngology-Head and Neck Surgery, Amsterdam UMC location Vrije Universiteit Amsterdam, De Boelelaan 1117, Amsterdam, The Netherlands; 2 Cancer Center Amsterdam, Treatment and Quality of Life, Amsterdam, The Netherlands; 3 Department Clinical, Neuro and Developmental Psychology, Vrije Universiteit Amsterdam, Van der Boechorststraat 7-9, Amsterdam, The Netherlands; 4 Amsterdam Public Health, Mental Health, Amsterdam, The Netherlands; 5 Department of Otorhinolaryngology, Erasmus MC Cancer Institute, University Medical Center, Rotterdam, The Netherlands; 6 Department of Physiology, Radboud University Medical Center, Nijmegen, The Netherlands; 7 Department Psychiatry, Amsterdam UMC location Vrije Universiteit Amsterdam, Amsterdam, The Netherlands; 8 Department of Radiation Oncology, University Medical Center Groningen, Groningen, The Netherlands; 9 Medical Psychology, Amsterdam UMC location University of Amsterdam, Meibergdreef 9, Amsterdam, the Netherlands; 10 Department of Otorhinolaryngology-Head and Neck Surgery, Radboud University Medical Center, Nijmegen, The Netherlands; 11 Department of Radiation Oncology, UMC Utrecht, Utrecht, The Netherlands; 12 Department of Epidemiology and Data Science, Amsterdam UMC location Vrije Universiteit Amsterdam, De Boelelaan, 1117 Amsterdam, The Netherlands

**Keywords:** Head and neck cancer, Health-related quality of life, Cortisol, Stress, Inflammation

## Abstract

**Purpose:**

The aim of this prospective cohort study was to estimate the relationship between the course of HRQOL in the first 2 years after diagnosis and treatment of head and neck cancer (HNC) and personal, clinical, psychological, physical, social, lifestyle, HNC-related, and biological factors.

**Methods:**

Data were used from 638 HNC patients of the NETherlands QUality of life and BIomedical Cohort study (NET-QUBIC). Linear mixed models were used to investigate factors associated with the course of HRQOL (EORTC QLQ-C30 global quality of life (QL) and summary score (SumSc)) from baseline to 3, 6, 12, and 24 months after treatment.

**Results:**

Baseline depressive symptoms, social contacts, and oral pain were significantly associated with the course of QL from baseline to 24 months. Tumor subsite and baseline social eating, stress (hyperarousal), coughing, feeling ill, and IL-10 were associated with the course of SumSc. Post-treatment social contacts and stress (avoidance) were significantly associated with the course of QL from 6 to 24 months, and social contacts and weight loss with the course of SumSc. The course of SumSc from 6 to 24 months was also significantly associated with a change in financial problems, speech problems, weight loss, and shoulder problems between baseline and 6 months.

**Conclusion:**

Baseline clinical, psychological, social, lifestyle, HNC-related, and biological factors are associated with the course of HRQOL from baseline to 24 months after treatment. Post-treatment social, lifestyle, and HNC-related factors are associated with the course of HRQOL from 6 to 24 months after treatment.

**Supplementary Information:**

The online version contains supplementary material available at 10.1007/s00520-023-07918-w.

## Introduction

There is convincing evidence that cancer patients have to deal with physical, psychological, and social side effects of the disease and its treatment, negatively affecting health-related quality of life (HRQOL) [1, 2]. In HNC patients, side effects such as oral dysfunction and related swallowing and speech impairment, also impact HRQOL [3–5]. HRQOL of HNC patients deteriorates before, during, and shortly after treatment, and generally improves from 6 months after treatment [6–12]. However, there is considerable variation between patients. Risk factors of poor HRQOL pertain to the disease and its treatment, and to personal, physical, psychological, social, and lifestyle factors [3–12]. Biological factors related to aging (telomere), stress (cortisol), and inflammation (cytokines) may also be associated with HRQOL [13–17]. Previous studies took only part of these factors into account [3–12, 18].

The aim of this prospective cohort study was to estimate the relationship between the course of HRQOL in the first 2 years after diagnosis and treatment of HNC and personal, clinical, psychological, physical, social, lifestyle, HNC-related, and biological factors. The results are important to better understand which baseline factors (how HNC patient enter the cancer trajectory) and factors assessed at 6 months after treatment (how they overcome the acute phase of treatment) are associated with the subsequent course of HRQOL over time.

## Materials and methods

### Patients and procedures

Data were used from the NETherlands QUality of life and BIomedical Cohort study (NET-QUBIC), a prospective cohort study among 739 HNC patients. NET-QUBIC comprises a Data Warehouse and Biobank integrating clinical information and data derived from patient-reported outcomes, fieldwork (interviews, functional tests), and biosamples [19, 20]. Recruitment took place between 2014 and 2018 in 5 of the 8 Dutch centers specialized in HNC. Inclusion criteria were newly diagnosed HNC (oral cavity, oropharynx, hypopharynx, larynx, unknown primary; all stages), age > 18, treatment with curative intent (all modalities), and able to write, read, and speak Dutch. Exclusion criteria were lymphoma, skin malignancies, or thyroid cancer; unable to understand the questions or test instructions; and severe psychiatric co-morbidities. Eligibility was assessed by the treating surgeon or radiation oncologist. The study protocol was approved by the medical ethical committee of VUmc (2013.301(A2018.307)-NL45051.029.13). All participants signed informed consent.

Details of NET-QUBIC are published previously: the study population (including retention and attrition), the electronic case report form (eCRF), the outcome assessment protocol, biobanking protocol, and data management (collection and storage) [18, 19]. In the current study, data was used from eCRF, patient-reported outcome measures (PROMs) (baseline, and 3, 6, 12, 24 months after finishing treatment), fieldwork assessments, and biobank sample collection (baseline and 6 months). The study population in the current study consisted of patients who completed the EORTC QLQ-C30 on at least one-time point.

### Sample size calculation

Sample size calculation was based on the research question of the entire NET-QUBIC study, i.e., to describe the course of HRQOL over time, with a difference over 60 months of 4 points change on QL between categories of relevant variables, using a residual standard deviation of 10 points within categories, and using an α of 0.05 and a power (β) of 0.80. For the dependency of the 5 repeated assessment points, we assumed an intraclass correlation coefficient of 0.50. This resulted in a total sample size of 462.

### Outcome assessment

Detailed descriptions of all outcome measures including references can be found in the NET-QUBIC data catalogue (https://researchers.kubusproject.nl/data-catalogue). A short description is provided below.

HRQOL was operationalized by the EORTC QLQ-C30 global quality of life subscale (QL) and the EORTC QLQ-C30 summary score (SumSC). QL is based on two items (global health status and quality of life). SumSC is based on five functional scales (physical, cognitive, emotional, social, role functioning), three symptom scales (fatigue, nausea/vomiting, pain), and five single items (dyspnea, insomnia, appetite loss, constipation, diarrhea). QL and SumSC scores range from 0 to 100. A higher score represents better HRQOL [20–22].

Personal factors included age (years), sex (men/ women), living status (alone/cohabiting), education (low/middle/high), personality (NEO Five Factor Inventory (NEO-FFI) scales; higher score indicating higher level of neuroticism, extraversion, openness to experience, agreeableness, or conscientiousness), coping style (Utrecht Coping List (UCL) scales; higher score indicating more active coping, palliative coping, avoidance coping, seeking support, passive coping, expression of emotions, and comforting thoughts), personal control (Pearlin Schooler and Mastery Scale (PSMS); higher score indicating higher level of mastery), and self-efficacy (Generalized Self-Efficacy Scale (GSE); higher score indicating better self-efficacy).

Clinical factors included tumor site (oral cavity, oropharynx, hypopharynx, larynx, unknown primary), stage (0–II/III–IV), treatment (single/multimodal), and WHO performance status (normal (ECOG score 0)/restricted (ECOG score > 0)). Oropharyngeal tumors were tested for HPV (negative/positive)). Comorbidity was assessed via the Adult Comorbidity Evaluation-27 Index (none/mild/moderate/severe). A history of a major depression disorder (yes/no) was assessed via the WHO-Composite International Diagnostic Interview (CIDI).

Physical factors included daily living (Instrumental Activities Daily Life (IADL); higher total score indicating less dependence), systolic and diastolic blood pressure, mean arterial blood pressure, heart rate, muscle strength of the upper extremity (hand grip test), and nutritional status (Mini Nutritional Assessment (MNA) categorized into malnourishment/risk of malnourishment/normal status).

Psychological factors included distress (Hospital Anxiety and Depression Scale (HADS total score), anxiety (HADS-A), and depressive symptoms (HADS-D); higher scores indicating more distress, anxiety, or depressive symptoms), fear of cancer recurrence (Cancer Worry Scale (CWS); higher score indicating more fear of recurrence), fatigue (Multidimensional Fatigue Inventory (MFI) scales; higher scores indicating more general fatigue, physical fatigue, reduced activity, reduced motivation, mental fatigue), sleep quality (Pittsburgh Sleep Quality Index (PSQI); higher total score indicating worse sleep quality); cognitive functioning (Cognitive Failures Questionnaire (CFQ); higher total score indicating more cognitive failures), adjustment to cancer (Mental Adjustment to Cancer scale (MAC) summary scores on positive and negative adjustment; higher scores indicating more positive adjustment or negative adjustment.

Social factors included social support (Social Support List - Interactions (SSL-I12); higher total score indicating better social support), trouble with social contacts and social eating (EORTC QLQ-HN35 subscales; higher score indicating more trouble), loneliness (Loneliness Scale de Jong Gierveld, higher total score indicating more loneliness), financial problems (EORTC QLQ-C30 item; higher score indicating more problems), and work (single item: no paid work/paid work).

Lifestyle factors included smoking (yes/no), excessive alcohol consumption (yes/no), body composition (body mass index (BMI)), physical activity (Physical Activity for the Elderly (PASE); higher total score indicating more physical activity), and stress (Impact of Event Scale-Revised (IES-R) scales; higher score indicating more intrusion, avoidance, hyperarousal, numbing, or sleep disturbance).

HNC-related factors were measured using the EORTC QLQ-HN35 scales; higher score indicates worse pain, swallowing, senses, speech, sexuality, problems with teeth, opening mouth, dry mouth, sticky saliva, coughing, feeling ill, more use of pain killers, nutritional supplements, feeding tube, weight loss, and weight gain. Shoulder function was measured using the Shoulder Disability Questionnaire (SDQ); higher total score indicates more shoulder problems. Hearing was measured using the Caron Questionnaire (Caron); higher total score indicates more hearing problems.

Biomarkers were measures of aging (leukocyte telomere length (LTL), stress (cortisol diurnal slope), and inflammation (pro-inflammatory cytokines Interleukin-6 (IL-6) (mg/ml), tumor necrosis factor-alpha (TNF-α) (pg/ml), acute phase protein C-reactive protein (CRP) (mg/ml)), and anti-inflammatory cytokine Interleukin-10 (IL-10) (pg/ml)). Telomere sequence copy number (T) in each sample was compared to a single-copy gene copy number (S), relative to a reference sample, where the resulting T/S ratio is proportional to mean LTL. Cortisol diurnal slope was calculated by subtracting cortisol level at 22:00 from cortisol level at awakening, divided by the duration (in hours) between these two time-points. A higher slope value means a steeper decline of cortisol, a lower slope value means a slower decline, and a negative slope value indicates an increasing cortisol level during the day.

### Statistical analysis

Baseline characteristics of included patients were compared with those not included (independent samples *t*-test for normally distributed continuous variables, Mann-Whitney *U* test for non-normally distributed variables, chi-square test for categorical variables). In these comparisons, *p*-values < 0.05 were considered statistically significant. Linear mixed model analysis (LMM) with fixed effect for time (categorical) and random effect for subject were used to investigate changes over time in the scores of QL and SumSc. *P*-values < 0.01 were considered statistically significant for the LMM analyses.

LMM was also used to assess whether variables related to personal, clinical, psychological, physical, social, lifestyle, HNC-related, and biological factors were associated with changes over time of QL and SumSc. The course of HRQOL from baseline to 24 months was investigated in relation to all factors assessed at baseline. The course of HRQOL from 6 to 24 months was investigated in two ways: first, in relation to baseline (static) personal and clinical factors, and other (dynamic) factors (psychological, clinical, physical, social, lifestyle, HNC-related, and biological factor) assessed at 6 months; second, in relation to the baseline factors and the change from baseline to 6 months in the dynamic factors. The models included fixed effects for time, the variable and their two-way interaction, and a random effect for subject. First, univariable analyses were carried out within each domain (personal, clinical, psychological, physical, social, lifestyle, HNC-related, and biological factors). Second, those factors that were significantly (*p*-value < 0.10) associated with the course of HRQOL in the univariable analyses were then entered into a multivariable model per domain. Those factors that remained significantly (*p* < 0.05) associated with the course over time were then entered in the overall multivariable model. In these final models, a *p*-value < 0.01 was considered statistically significant. Estimated QL and SumSc at each of the assessment points were plotted for the significant variables in these final models, at low (around the 25th percentile), moderate (around the median) and high (75th percentile) values of the different variables.

All statistical analyses were conducted using the IBM Statistical Package for the Social Science (SPSS) version 26 (IBM Corp., Armonk, NY, USA; 2018).

## Results

### Study population

**Fig. 1:**
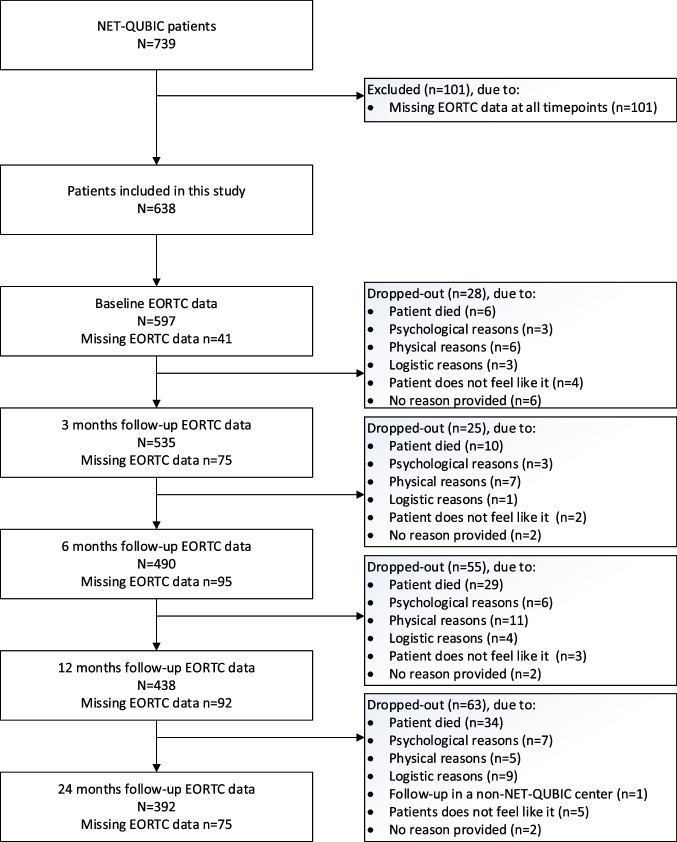
Flow diagram of the study

Of the 739 participants in NET-QUBIC, 638 (86%) were included. Included patients more often lived together, had a higher education level, had more often a lower tumor stage, a HPV positive oropharynx tumor, and a lower level of comorbidity (*p* < 0.05) (Table 1). Table 1 provides an overview of the study population on all personal and clinical factors. The flow diagram of the study including reasons for drop-out is presented in Fig. 1.

**Table 1:** Overview of the study population

	Excluded* (*n* = 101)		Included (*n* = 638)		*p*-value**
	*N*	%	*N*	%	
Personal factors					
Age (mean (SD))	101	61.9 (11.2)	638	63.5 (9.5)	0.19
Sex		%		%	0.80
*Male*	74	73.3	475	74.5	
*Female*	27	26.7	163	25.5	
Living situation				%	< 0.000
*Living together*	30	29.7	455	71.3	
*Living alone*	31	30.7	133	20.8	
*Missing*	40	39.6	50	7.8	
Highest level of education					0.022
*Low*	35	34.7	244	38.2	
*Middle*	16	15.8	155	24.3	
*High*	10	9.9	188	29.5	
*Missing*	40	39.6	51	8.0	
Clinical factors					
Tumor subsite					0.13
*Oral cavity*	21	20.8	178	27.9	
*Oropharynx*	38	37.6	224	35.1	
*Hypopharynx*	12	11.9	40	6.3	
*Larynx*	28	27.7	177	27.7	
*Unknown primary*	2	2.0	19	3.0	
Tumor stage					0.02
*0/I*	11	10.9	152	23.8	
*II*	17	16.8	115	18.0	
*III*	22	21.8	105	16.5	
*IVA/IVB/IVC*	51	50.5	266	41.7	
HPV status (oropharynx)					0.04
*Negative*	30	29.7	134	21.0	
*Positive*	14	13.9	127	19.9	
*Missing*	57	56.4	377	59.1	
Treatment modality					0,47
*Single treatment*	50	49.5	343	53.8	
*Multiple treatment*	50	49.5	294	46.1	
*Missing*	1	1.0	1	0.2	
Comorbidity (ACE-27)					0.02
*None*	17	16.8	187	29.3	
*Mild*	31	30.7	233	36.5	
*Moderate*	29	28.7	126	19.7	
*Severe*	13	12.9	63	9.9	
*Missing*	11	10.9	29	4.5	
Major depression—in lifetime					0.85
*No*	50	49.5	429	67.2	
*Yes*	10	9.9	80	12.5	
*Missing*	41	40.6	129	20.2	
WHO performance					0.09
*ECOG score 0*	62	61.4	445	69.7	
*ECOG score > 0*	39	38.6	193	30.3	

*No EORTC QLQ-C30 data available

**Missings not in the statistical analyses

Abbreviations: *SD* standard deviation, *HPV* human papilloma virus, *WHO* World Health Organization

### HRQOL over time and associated factors

There was a significant (*p* < 0.01) change from baseline to 24 months in QL, with the lowest scores at baseline and 3 months after treatment after which QL improved (Table 2). The change in QL from 6 to 24 months was not statistically significant (*p* = 0.032) (Table 2). The change in the course of SumSc from baseline to 24 months was significant (*p* < 0.01), with a decline from baseline to 3 months after which SumSc improved (Table 2). The change in SumSc from 6 to 24 months was not statistically significant (*p* = 0.02) (Table 2).

**Table 2:** The course of health-related quality of life over time

Measurement	*N*	Observed data					Estimated with LMM			*p*-value	*p*-value compared to T0/M6
		Mean	SD	Median	Interquartile range		Mean	95% CI			
EORTC—global quality of life											
T0:baseline	596	71.7	19.0	75.0	58.3	83.3	71.6	70.2	73.0	0.000	
3 months	535	73.8	17.6	75.0	66.7	83.3	73.5	72.0	75.0		0.013
6 months	490	76.7	17.5	83.3	66.7	83.3	75.8	74.2	77.3		0.000
12 months	437	79.5	17.0	83.3	66.7	91.7	77.9	76.3	79.5		0.000
24 months	392	78.8	16.9	83.3	66.7	91.7	76.7	75.1	78.4		0.000
6 months	490	76.7	17.5	83.3	66.7	83.3	76.7	75.2	78.2	0.032	
12 months	437	79.5	17.0	83.3	66.7	91.7	78.7	77.2	80.3		0.009
24 months	392	78.8	16.9	83.3	66.7	91.7	77.8	76.1	79.4		0.195
EORTC—summary score											
T0:baseline	592	86.4	11.9	89.8	80.6	95.5	86.2	85.2	87.2	0.000	
3 months	527	83.6	13.4	87.0	75.9	93.7	83.1	82.1	84.2		0.000
6 months	486	86.8	11.9	89.3	81.7	95.8	86.1	85.1	87.2		0.85
12 months	436	88.9	11.4	91.9	84.9	97.4	87.5	86.4	88.6		0.010
24 months	391	88.7	10.8	91.7	83.9	96.4	87.1	86.0	88.2		0.10
6 months	486	86.8	11.9	89.3	81.7	95.8	86.8	85.8	87.9	0.020	
12 months	436	88.9	11.4	91.9	84.9	97.4	88.1	87.1	89.2		0.007
24 months	391	88.7	10.8	91.7	83.9	96.4	87.8	86.7	88.8		0.059

Results of univariable and multivariable LMM analyses within each domain and multivariable LMM analyses across all domains are presented in Tables 3 and 4. The variables that were significantly associated with QL or SumSc over time in the overall multivariable models are illustrated in Appendix A.

**Table 3:** Associations between baseline and post-treatment factors and the course of the EORTC QLQ-C30 global quality of life subscale (QL) during 24-month follow-up

	Baseline factor (T0)			Post-treatment factor (M6)			Change in factor M6-T0		
	*p*-value univariable within domain	*p*-value multivariable within domain	*p*-value over all domains	*p*-value univariable within domain	*p*-value multivariable within domain	*p*-value over all domains	*p*-value univariable within domain	*p*-value multivariable within domain	*p*-value over all domains
Personal factors									
Age	0.08	0.03	n.s.	0.39	n.i.	n.i.			
Sex	0.11	n.i.	n.i.	0.73	n.i.	n.i.			
Living situation	0.75	n.i.	n.i.	0.87	n.i.	n.i.			
Educational level	0.93	n.i.	n.i.	0.61	n.i.	n.i.			
Personality (NEO-FFI)									
Neuroticism	0.11	n.i.	n.i.	0.59	n.i.	n.i.			
Extraversion	0.57	n.i.	n.i.	0.83	n.i.	n.i.			
Openness	0.35	n.i.	n.i.	0.64	n.i.	n.i.			
Agreeableness	0.22	n.i.	n.i.	0.21	n.i.	n.i.			
Conscientiousness	0.61	n.i.	n.i.	0.58	n.i.	n.i.			
Coping (UCL)									
Active coping	0.06	n.s.	n.i.	0.06	n.s.	n.i.			
Palliative coping	0.05	n.s.	n.i.	0.03	0.03	n.s.			
Avoidance coping	< 0.001	n.s.	n.i.	0.41	n.i.	n.i.			
Seeking support	0.11	n.i.	n.i.	0.04	n.s.	n.i.			
Passive coping	0.01	0.01	n.s.	0.19	n.i.	n.i.			
Expression of emotions	0.65	n.i.	n.i.	0.94	n.i.	n.i.			
Comforting thoughts	0.12	n.i.	n.i.	0.42	n.i.	n.i.			
Personal control (PSMS)	0.28	n.i.	n.i.	0.87	n.i.	n.i.			
Self-efficacy (GSE)	< 0.001	n.s.	n.i.	0.96	n.i.	n.i.			
Clinical factors									
Tumor subsite	0.04	0.04	n.s.	0.61	n.i.	n.i.			
Tumor stage	0.48	n.i.	n.i.	0.34	n.i.	n.i.			
HPV status	0.36	n.i.	n.i.	0.09	n.s.	n.i.			
Treatment modality	0.34	n.i.	n.i.	0.17	n.i.	n.i.			
Comorbidity	0.59	n.i.	n.i.	0.93	n.i.	n.i.			
Major depression—in lifetime	0.69	n.i.	n.i.	0.56	n.i.	n.i.			
WHO performance	0.86	n.i.	n.i.	0.79	n.i.	n.i.			
Physical factors									
Daily activities (IADL)	0.18	n.i.	n.i.	0.50	n.i.	n.i.	0.39	n.i.	n.i.
Systolic blood pressure	0.38	n.i.	n.i.	0.93	n.i.	n.i.	0.94	n.i.	n.i.
Diastolic blood pressure	0.18	n.i.	n.i.	0.61	n.i.	n.i.	0.86	n.i.	n.i.
Mean arterial blood pressure	0.23	n.i.	n.i.	0.74	n.i.	n.i.	0.89	n.i.	n.i.
Heart rate	0.71	n.i.	n.i.	0.97	n.i.	n.i.	0.93	n.i.	n.i.
Muscle strength (hand grip test)	0.02	< 0.001	n.s.	0.44	n.i.	n.i.	0.12	n.i.	n.i.
Nutritional status (MNA)	< 0.0001	< 0.0001	n.s.	0.18	n.i.	n.i.	0.27	n.i.	n.i.
Psychological factors									
Anxiety (HADS-A)	< 0.001	n.s.	n.i.	0.22	n.i.	n.i.	0.85	n.i.	n.i.
Depression (HADS-D)	< 0.001	< 0.001	< 0.001	0.07	n.s	n.i.	0.62	n.i.	n.i.
Distress (HADS Total)	< 0.001	n.i.	n.i.	0.09	n.s	n.i.	0.74	n.i.	n.i.
Fear of recurrence (CWS)	< 0.001	0.04	n.s.	0.04	0.04	n.s.	0.02	0.02	n.s.
Fatigue (MFI)	< 0.001								
General fatigue	< 0.001	n.s.	n.i.	0.05	n.s	n.i.	0.87	n.i.	n.i.
Physical fatigue	0.001	n.s.	n.i.	0.08	n.s	n.i.	0.05	n.s.	n.i.
Reduced activity	0.001	n.s.	n.i.	0.14	n.i.	n.i.	0.38	n.i.	n.i.
Reduced motivation	< 0.001	n.s.	n.i.	0.14	n.i.	n.i.	0.23	n.i.	n.i.
Mental fatigue	0.001	n.s.	n.i.	0.13	n.i.	n.i.	0.78	n.i.	n.i.
Sleep quality (PSQI)	<0.001	n.s.	n.i.	0.12	n.i.	n.i.	0.20	n.i.	n.i.
Cognitive function (CFQ)	0.03	n.s.	n.i.	0.15	n.i.	n.i.	0.41	n.i.	n.i.
Adjustment to cancer									
Positive adjustment (MAC)	0.48	n.i.	n.i.	0.45	n.i.	n.i.	0.21	n.i.	n.i.
Negative adjustment (MAC)	0.000	n.s.	n.i.	0.04	n.s	n.i.	0.22	n.i.	n.i.
Social factors									
Social support (SSL-I12)	0.16	n.i.	n.i.	0.12	n.i.	n.i.	0.03	0.03	n.s.
Social contact (EORTC HN35)	< 0.001	0.01	0.008	< 0.001	< 0.001	0.001	0.02	n.s.	n.i.
Social eating (EORTC HN35)	< 0.001	< 0.001	n.s.	0.79	n.i.	n.i.	0.03	n.s.	n.i.
Loneliness (de Jong Gierveld)	0.23	n.i.	n.i.	0.63	n.i.	n.i.	0.43	n.i.	n.i.
Financial problems (EORTC-HN35)	0.41	n.i.	n.i.	0.08	n.s	n.i.	0.01	n.s.	n.i.
Work (single item)	0.51	n.i.	n.i.	0.26	n.i.	n.i.			
Biomarkers									
Telomere length	0.89	n.i.	n.i.	0.56	n.i.	n.i.			
Cortisol diurnal slope	0.36	n.i.	n.i.	0.59	n.i.	n.i.	0.91	n.i.	n.i.
IL-6	0.18	n.i.	n.i.	0.10	n.i.	n.i.	0.94	n.i.	n.i.
IL-10	0.91	n.i.	n.i.	0.47	n.i.	n.i.	0.63	n.i.	n.i.
CRP	0.40	n.i.	n.i.	0.42	n.i.	n.i.	0.73	n.i.	n.i.
TNF-α	0.24	n.i.	n.i.	0.18	n.i.	n.i.	0.52	n.i.	n.i.
Lifestyle factors									
Smoking	0.15	n.i.	n.i.	0.10	n.i.	n.i.	0.89	n.i.	n.i.
Alcohol consumption	0.72	n.i.	n.i.	0.34	n.i.	n.i.	0.72	n.i.	n.i.
Body mass index	0.86	n.i.	n.i.	1.00	n.i.	n.i.	0.73	n.i.	n.i.
Physical activity (PASE)	0.78	n.i.	n.i.	0.32	n.i.	n.i.	0.33	n.i.	n.i.
Stress (IES-R)									
Intrusion	< 0.001	< 0.001	n.s.	0.19	n.i.	n.i.	0.05	n.s	n.i.
Avoidance	<0.001	n.s.	n.i.	< 0.001	< 0.001	0.006	0.63	n.i.	n.i.
Hyperarousal	< 0.001	n.s.	n.i.	0.92	n.i.	n.i.	0.02	0.02	0.017
Numbing	< 0.001	n.s.	n.i.	0.76	n.i.	n.i.	0.34	n.i.	n.i.
Sleep disturbance	< 0.001	n.s.	n.i.	0.90	n.i.	n.i.	0.09	n.s	n.i.
HNC related factors									
EORTC QLQ HN35									
Pain	< 0.001	< 0.001	0.005	0.01	0.01	n.s.	0.01	n.s	n.i.
Swallowing	< 0.001	n.s.	n.i.	0.71	n.i.	n.i.	0.08	n.s	n.i.
Senses	< 0.001	0.042	n.s.	0.46	n.i.	n.i.	0.71	n.i.	n.i.
Speech	< 0.001	n.s.	n.i.	0.57	n.i.	n.i.	0.08	n.s	n.i.
Sexuality and intimacy	< 0.001	n.s.	n.i.	0.94	n.i.	n.i.	0.67	n.i.	n.i.
Teeth	< 0.001	n.s.	n.i.	0.63	n.i.	n.i.	0.91	n.i.	n.i.
Opening mouth	< 0.001	n.s.	n.i.	0.74	n.i.	n.i.	0.63	n.i.	n.i.
Dry mouth	0.04	n.s.	n.i.	0.31	n.i.	n.i.	0.19	n.i.	n.i.
Sticky saliva	0.001	n.s.	n.i.	0.44	n.i.	n.i.	0.52	n.i.	n.i.
Coughing	0.51	n.i.	n.i.	0.08	n.s.	n.i.	0.10	n.i.	n.i.
Feeling ill	< 0.001	< 0.001	n.s.	0.01	0.03	n.s.	0.00	0.02	n.s.
Painkillers	< 0.001	n.s.	n.i.	0.18	n.i.	n.i.	0.60	n.i.	n.i.
Nutritional supplements	0.25	n.i.	n.i.	0.46	n.i.	n.i.	0.82	n.i.	n.i.
Feeding tube	0.06	n.s.	n.i.	0.35	n.i.	n.i.	0.48	n.i.	n.i.
Weight loss	< 0.001	n.s.	n.i.	0.01	< 0.001	n.s.	0.01	0.03	n.s.
Weight gain	0.74	n.i.	n.i.	0.55	n.i.	n.i.	0.93	n.i.	n.i.
Shoulder function (SDQ)	0.52	n.i.	n.i.	0.02	n.s	n.i.	0.05	0.03	n.s.
Hearing (Caron Questionnaire)	0.07	n.s.	n.i.	0.42	n.i.	n.i.	0.12	n.i.	n.i.

Abbreviations: *T0* baseline, *M6* 6 months after treatment, *n.i.* not included in the multivariate model, *n.s.* not significant, *NEO-FFI* NEO Five Factor Inventory, *UCL* Utrecht Coping List, *PSMS* Pearlin Scoolar and Mastery Scale, *GSE* Generalized Self-Efficacy scale, *HPV* human papilloma virus, *WHO* World Health Organization, *IADL* Instrumental Activities Daily Life, *MNA* Mini Nutritional Assessment, *HADS* Hospital Anxiety and Depression Scale, *CWS* Cancer Worry Scale, *MFI* Multidimensional Fatigue Scale, P*SQI* Pittsburgh Sleep Quality Index, *CFQ* Cogntive Failures Questionnaire, *MAC* Mental Adjustment to Cancer, *SSL-I12* Social Support List – Interactions, *EORTC HN35* European Organization for Research and Treatment of Cancer Head and Neck 35 Module, *IL-6* Interleukin-6, *IL-10* Interleukin-10, *TNF-α* tumor necrosis factor-alpha, *CRP* C-reactive protein, *PASE* Physical Activity Scale for the Elderly, *IES-R* Impact of Event Scale-Revised, *SDQ* Shoulder Disability Questionnaire

**Table 4:** Overview the EORTC QLQ-C30 Summary Score (SumSc) and associated factors

	T0			M6			Change M6-T0		
	*p*-value univariable within domain	*p*-value multivariable within domain	*p*-value over all domains	*p*-value univariable within domain	*p*-value multivariable within domain	*p*-value over all domains	*p*-value univariable within domain	*p*-value multivariable within domain	*p*-value over all domains
Personal factors									
Age	0.009	0.009	n.s.	0.19	n.i.	n.i.			
Sex	0.079	n.s.	n.i.	0.36	n.i.	n.i.			
Living situation	0.17	n.i.	n.i.	0.041	n.s.	n.i.			
Educational level	0.84	n.i.	n.i.	0.52	n.i.	n.i.			
Personality (NEO-FFI)									
Neuroticism	0.020	n.s.	n.i.	0.13	n.i.	n.i.			
Extraversion	0.26	n.i.	n.i.	0.18	n.i.	n.i.			
Openness	0.44	n.i.	n.i.	0.89	n.i.	n.i.			
Agreeableness	0.53	n.i.	n.i.	0.28	n.i.	n.i.			
Conscientiousness	0.75	n.i.	n.i.	0.96	n.i.	n.i.			
Coping (UCL)									
Active coping	0.50	n.i.	n.i.	0.66	n.i.	n.i.			
Palliative coping	0.039	n.s.	n.i.	0.47	n.i.	n.i.			
Avoidance coping	0.001	n.s.	n.i.	0.20	n.i.	n.i.			
Seeking support	0.033	n.s.	n.i.	0.66	n.i.	n.i.			
Passive coping	< 0.001	< 0.001	n.s.	0.009	0.009	n.s.			
Expression of emotions	0.14	n.i.	n.i.	0.44	n.i.	n.i.			
Comforting thoughts	0.047	n.s.	n.i.	0.54	n.i.	n.i.			
Personal control (PSMS)	0.19	n.i.	n.i.	0.070	n.s.	n.i.			
Self-efficacy (GSE)	0.060	n.s.	n.i.	0.49	n.i.	n.i.			
Clinical factors									
Tumor subsite	< 0.001	< 0.001	0.002	0.010	0.004	n.s.			
Tumor stage	0.029	n.s.	n.i.	0.59	n.i.	n.i.			
HPV status	0.46	n.i.	n.i.	0.54	n.i.	n.i.			
Treatment modality	0.068	n.s.	n.i.	0.47	n.i.	n.i.			
Comorbidity	0.002	0.001	n.s.	0.026	0.016	n.s.			
Major depression—in lifetime	0.46	n.i.	n.i.	0.26	n.i.	n.i.			
WHO performance	0.13	n.i.	n.i.	0.014	n.s.	n.i.			
Physical factors									
Daily activities (IADL)	0.91	n.i.	n.i.	0.67	n.i.	n.i.	0.57	n.i.	n.i.
Systolic blood pressure	0.055	n.s.	n.i.	0.78	n.i.	n.i.	0.94	n.i.	n.i.
Diastolic blood pressure	0.58	n.i.	n.i.	0.10	n.i.	n.i.	0.95	n.i.	n.i.
Mean arterial blood pressure	0.32	n.i.	n.i.	0.32	n.i.	n.i.	0.94	n.i.	n.i.
Heart rate	0.52	n.i.	n.i.	0.61	n.i.	n.i.	0.46	n.i.	n.i.
Muscle strength (hand grip test)	0.43	n.i.	n.i.	0.56	n.i.	n.i.	0.15	n.i.	n.i.
Nutritional status (MNA)	0.007	0.007	n.s.	0.11	n.i.	n.i.	0.021	0.021	n.s
Psychological factors									
Anxiety (HADS-A)	0.000	n.s.	n.i.	0.002	n.s.	n.i.	0.73	n.i.	n.i.
Depression (HADS-D)	0.000	0.000	n.s.	0.000	0.000	n.s.	0.008	n.s	n.i.
Distress (HADS Total)	0.000	n.i.	n.i.	0.000	n.i.	n.i.	0.11	n.i.	n.i.
Fear of recurrence (CWS)	0.000	n.s.	n.i.	0.10	n.i.	n.i.	0.23	n.i.	n.i.
Fatigue (MFI)									
General fatigue	0.000	n.s.	n.i.	0.000	n.s.	n.i.	0.067	n.s	n.i.
Physical fatigue	0.001	n.s.	n.i.	0.000	n.s.	n.i.	0.000	0.000	n.s
Reduced activity	0.000	n.s.	n.i.	0.016	n.s.	n.i.	0.86	n.i.	n.i.
Reduced motivation	0.000	n.s.	n.i.	0.004	n.s.	n.i.	0.14	n.i.	n.i.
Mental fatigue	0.000	n.s.	n.i.	0.000	n.s.	n.i.	0.15	n.i.	n.i.
Sleep quality (PSQI)	0.002	n.s.	n.i.	0.008	n.s.	n.i.	0.29	n.i.	n.i.
Cognitive function (CFQ)	0.015	n.s.	n.i.	0.007	n.s.	n.i.	0.53	n.i.	n.i.
Adjustment to cancer									
Positive adjustment (MAC)	0.84	n.i.	n.i.	0.69	n.i.	n.i.	0.83	n.i.	n.i.
Negative adjustment (MAC)	0.013	n.s.	n.i.	0.001	n.s.	n.i.	0.061	n.s	n.i.
Social factors									
Social support (SSL-I12)	0.091	n.s.	n.i.	0.76	n.i.	n.i.	0.042	0.018	n.s
Social contact (EORTC HN35)	0.000	n.s.	n.i.	0.000	0.000	0.000	0.000	n.s.	n.i.
Social eating (EORTC HN35)	0.000	0.000	0.009	0.000	n.s.	n.i.	0.003	0.000	n.s
Loneliness (de Jong Gierveld)	0.38	n.i.	n.i.	0.23	n.i.	n.i.	0.94	n.i.	n.i.
Financial problems (EORTC-HN35)	0.003	n.s.	n.i.	0.003	n.s.	n.i.	0.000	0.000	0.007
Work (single item)	0.27	n.i.	n.i.	0.70	n.i.	n.i.			
Biomarkers									
Telomere length	0.85	n.i.	n.i.	0.54	n.i.	n.i.			
Cortisol diurnal slope	0.82	n.i.	n.i.	0.22	n.i.	n.i.	0.46	n.i.	n.i.
IL-6	0.081	n.s.	n.i.	0.53	n.i.	n.i.	0.99	n.i.	n.i.
IL-10	0.013	0.013	0.002	0.67	n.i.	n.i.	0.76	n.i.	n.i.
CRP	0.30	n.i.	n.i.	0.69	n.i.	n.i.	0.22	n.i.	n.i.
TNF-α	0.21	n.i.	n.i.	0.23	n.i.	n.i.	0.87	n.i.	n.i.
Lifestyle factors									
Smoking	0.14	n.i.	n.i.	0.20	n.i.	n.i.	0.79	n.i.	n.i.
Alcohol consumption	0.33	n.i.	n.i.	0.057	n.s.	n.i.	0.74	n.i.	n.i.
Body Mass Index	0.56	n.i.	n.i.	0.74	n.i.	n.i.	0.69	n.i.	n.i.
Physical activity (PASE)	0.84	n.i.	n.i.	0.039	0.039	n.s.	0.081	n.s.	n.i.
Stress (IES-R)									
Intrusion	0.000	n.s.	n.i.	0.53	n.i.	n.i.	0.051	0.051	n.i.
Avoidance	0.000	n.s.	n.i.	0.084	n.s.	n.i.	0.88	n.i.	n.i.
Hyperarousal	0.000	0.000	0.000	0.45	n.i.	n.i.	0.083	n.s.	n.i.
Numbing	0.000	n.s.	n.i.	0.48	n.i.	n.i.	0.45	n.i.	n.i.
Sleep disturbance	0.000	n.s.	n.i.	0.074	n.s.	n.i.	0.63	n.i.	n.i.
HNC related factors									
EORTC QLQ HN35									
Pain	0.000	n.s.	n.i.	0.000	n.s.	n.i.	0.014	n.s.	n.i.
Swallowing	0.046	n.s.	n.i.	0.000	n.s.	n.i.	0.20	n.s.	n.i.
Senses	0.001	n.s.	n.i.	0.20	n.i.	n.i.	0.23	n.i.	n.i.
Speech	0.001	0.018	n.s.	0.000	0.008	n.s.	0.000	0.006	0.002
Sexuality and intimacy	0.000	n.s.	n.i.	0.29	n.i.	n.i.	0.79	n.i.	n.i.
Teeth	0.000	n.s.	n.i.	0.049	n.s.	n.i.	0.37	n.i.	n.i.
Opening mouth	0.000	0.000	n.s.	0.34	n.i.	n.i.	0.45	n.i.	n.i.
Dry mouth	0.030	n.s.	n.i.	0.038	n.s.	n.i.	0.35	n.i.	n.i.
Sticky saliva	0.014	n.s.	n.i.	0.041	n.s.	n.i.	0.42	n.i.	n.i.
Coughing	0.019	0.000	0.000	0.004	n.s.	n.i.	0.063	n.s.	n.i.
Feeling ill	0.000	0.000	0.000	0.000	0.013	n.s.	0.000	0.001	n.s.
Painkillers	0.075	n.s.	n.i.	0.001	n.s.	n.i.	0.13	n.i.	n.i.
Nutritional supplements	0.16	n.i.	n.i.	0.056	n.s.	n.i.	0.54	n.i.	n.i.
Feeding tube	0.22	n.i.	n.i.	0.089	n.s.	n.i.	0.28	n.i.	n.i.
Weight loss	0.012	n.s.	n.i.	0.000	0.001	0.000	0.000	0.000	0.000
Weight gain	0.80	n.i.	n.i.	0.39	n.i.	n.i.	0.66	n.i.	n.i.
Shoulder function (SDQ)	0.75	n.i.	n.i.	0.000	0.016	n.s.	0.000	0.000	0.001
Hearing (CARON)	0.82	n.i.	n.i.	0.68	n.i.	n.i.	0.093	n.s.	n.i.

Abbreviations: *T0* baseline, *M6* 6 months after treatment, *n.i.* not included in the multivariate model, *n.s.* not significant, *NEO-FFI* NEO Five Factor Inventory, *UCL* Utrecht Coping List, *PSMS* Pearlin Scoolar and Mastery Scale, *GSE* Generalized Self-Efficacy scale, *HPV* human papilloma virus, *WHO* World Health Organization, *IADL* Instrumental Activities Daily Life, *MNA* Mini Nutritional Assessment, *HADS* Hospital Anxiety and Depression Scale, *CWS* Cancer Worry Scale, *MFI* Multidimensional Fatigue Scale, P*SQI* Pittsburgh Sleep Quality Index, *CFQ* Cogntive Failures Questionnaire, *MAC* Mental Adjustment to Cancer, *SSL-I12* Social Support List – Interactions, *EORTC HN35* European Organization for Research and Treatment of Cancer Head and Neck 35 Module, *IL-6* Interleukin-6, *IL-10* Interleukin-10, *TNF-α* tumor necrosis factor-alpha, *CRP* C-reactive protein, *PASE* Physical Activity Scale for the Elderly, *IES-R* Impact of Event Scale-Revised, *SDQ* Shoulder Disability Questionnaire

In the course of HRQOL from baseline to 24 months, the overall multivariable analyses (Table 3) showed that the course of QL was significantly associated with depressive symptoms, social contacts, and pain at baseline. The course of SumSc from baseline to 24 months was significantly associated with tumor subsite, social eating, stress (hyperarousal), coughing, feeling ill, and IL-10, as measured at baseline. Regarding tumor subsite, there were distinct courses of SumSc over time (Appendix A1). Based on the other figures in Appendix A1, several patterns were observed: (a) patients with more depressive symptoms, less social contacts, or more oral pain at baseline had a relatively smaller deterioration in QL from baseline to 3 months and a larger or similar improvement afterwards approaching the level of patients with less of these symptoms at baseline; (b) patients with more problems with social eating or feeling ill at baseline had a relatively larger improvement of SumSc from baseline to 6 months and similar deterioration afterwards; (c) patients with more stress, more coughing, or a higher level of IL10 at baseline had a relatively larger deterioration in SumSc from baseline to 3 months and similar improvement from 6 to 24 months.

In the course of HRQOL from 6 to 24 months, the overall multivariable analyses (Tables 3 and 4) showed that the course of QL was significantly associated with social contacts and stress (avoidance) as measured at 6 months. The course of SumSc was significantly associated with social contacts and weight loss at 6 months. Based on the figures in Appendix A2, several patterns were observed: (a) patients with less problems with social contacts at 6 months after treatment had a relatively smaller improvement in QL from 6 to 12 months but no deterioration from 12 to 24 months; (b) patients with less social contacts at 6 months had a relatively larger improvement in SumSc from 6 to 12 months and no deterioration from 12 to 24 months; (c) patients with more stress at 6 months had a relatively larger improvement in QL from 6 to 12 months and a larger deterioration from 12 to 24 months; (d) patients with more weight loss at 6 months had a relatively larger deterioration in SumSc from 6 to 12 to 24 months. Furthermore, the course of SumSc from 6 to 24 months was significantly associated with a change in financial problems, speech problems, weight loss, and shoulder problems between baseline and 6 months (Tables 3 and 4). Again, various patterns were observed (Appendix A3): (a) patients with a worsening of financial problems, speech, and shoulder problems had a relatively larger improvement in SumSC between 6 and 12 months; (b) patients with a worsening of weight loss between baseline and 6 months had a relatively larger deterioration in SumSc between 6 and 24 months.

## Discussion

In this cohort of 638 HNC patients, there was a significant change of HRQOL over time with worse scores at baseline and 3 months after treatment. The absolute changes over time in QL (maximal improvement ≤ 7.8) were small, considering previous reported minimally important difference (MID) values of 8.64 on the EORTC QLQ-C30 QL scale among HNC patients [23]. There are no MID values available for SumSc but mean differences over time in the current cohort were also small (≤ 5.3 on a scale of 100). HRQOL of cancer patients in the Netherlands is generally higher compared to other countries [24]. Mean QL scores in this HNC cohort ranged from 71.7 to 79.5 and seem not to be different from mean scores of 77.4 and 77.9 as found in Dutch reference data [24, 25]. The same holds for SumSc: 86.8 to 88.9 in this HNC cohort versus 93.1 in a Dutch reference population [25]. It can be concluded that, in general, HRQOL of this HNC cohort was relatively good.

The baseline and post-treatment status of HNC patients were significantly associated with the course of HRQOL over time. HRQOL of patients who had more psychosocial problems (depressive symptoms, social contacts, social eating), oral pain or who were feeling more ill at baseline deteriorated relatively less and/or improved more from baseline to 24 months after treatment. In contrast, HRQOL of patients with a higher level of IL10, more stress, or more coughing at baseline deteriorated relatively more. As to post-treatment status, HNC patients with worse social contacts, and more speech, shoulder, and financial problems at 6 months after treatment, had a relatively larger improvement in HRQOL from 6 to 24 months after treatment, while patients with more stress and weight loss at 6 months after treatment had a relatively larger deterioration in HRQOL. It may be that HNC patients with more psychosocial and functional problems at baseline or post-treatment were referred to supportive care (including psychosocial care, pain medication, physiotherapy, speech therapy, and nutritional advice), which is effective to improve such problems and improve HRQOL [3–5, 26–28]. A poorer health condition due to coughing, stress, or weight loss may be related to underlying medical illness such as HNC recurrence, a second primary tumor (e.g., in the lungs), cachexia, or hypothyroidism [16, 29]. The role of the anti-inflammatory cytokine IL-10 (as measured at baseline) is interesting but also puzzling. Tumor and immune cells are sources of cytokines, which can lead to various symptoms negatively influencing HRQOL. Stress can corroborate the production of pro-inflammatory cytokines [14, 30]. However, IL-6, TNF-α, and CRP were not significantly associated with the course of HRQOL in the current study. It might be that the high level of IL-10 represents the remain of earlier inflammation before or at baseline, but more research is needed.

The strengths of this study are that we used the validated EORTC QLQ-C30 QL and SumSc which reflect overall HRQOL. To handle missing data at random which is common in longitudinal studies investigating HRQOL, we applied LMM analyses with maximum likelihood estimation which account for missing data. We did not account for missing data not at random. A limitation is the use of assumption of a compound symmetry covariance structure in the LMM analyses. Future researchers may explore other covariance structures. Another limitation of this study is that the NET-QUBIC cohort is not completely representative for the Dutch HNC population regarding age, sex, tumor subsite, and treatment modality [19, 20]. Retention rates were high at 2 years follow-up (80% among HNC patients alive) [20] but there was some selection in terms of tumor stage, physical performance, comorbidity and age, which might limit the representativeness of the results of this study. Another limitation is that the large number of variables may have induced co-incidental findings.

It is striking that so many variables were not significantly associated with the course of HRQOL over time, including none of the personal factors (age, sex, living status, level of education, personality, coping style, personal control, and self-efficacy), clinical (tumor stage, treatment modality, performance status, HPV, comorbidity), physical (daily living, blood pressure, heart rate, muscle strength, nutritional status), psychological (distress, anxiety, fear of cancer recurrence, fatigue, sleep, cognitive functioning adjustment to cancer), and lifestyle factors (smoking, alcohol use, BMI, physical activity). That does not mean that these factors are not important in HNC research, for example when developing prediction models or symptom clusters. It might be worthwhile, dependent on the research question, to stratify for HNC subsite in future research since (slightly) different courses of HRQOL were observed. Also, standardization of outcomes measurements is needed, preferably in consensus with (inter)national HNC organizations.

From a clinical point of view, understanding which factors impact an individual’s quality of life can help healthcare providers for instance by developing a risk assessment tool in clinical practice and targeted interventions. Also, it would be interesting to evaluate if changes in HRQoL over time are associated with patients’ need for supportive care.

## Conclusion

Baseline clinical, psychological, social, lifestyle, HNC-related, and biological factors are associated with the course of HRQOL from baseline to 24 months after treatment. Post-treatment social, lifestyle, and HNC-related factors are associated with the course of HRQOL from 6 to 24 months after treatment.

## Supplementary information

ESM 1
