# Supplementary material for: The course of health-related quality of life in the first 2 years after a diagnosis of head and neck cancer: the role of personal, clinical, psychological, physical, social, lifestyle, disease-related, and biological factors
Source: Support Care Cancer. 2023 Jul 11;31(8):458. doi: 10.1007/s00520-023-07918-w (PMC10335953; doi:10.1007/s00520-023-07918-w)
Supplement: Supplementary file 1 — ESM 1 [file 520_2023_7918_MOESM1_ESM.docx]

Appendix A Illustrations of the course of QL and SuMsc over time in relation to various variables

Concerning the variables that were significantly associated with QL or SumSc over time in the overall multivariate models, the figures in Appendix A illustrate the course of QL and SumSc over time in relation to each variable.

A smaller deterioration and/or a larger improvement indicates a better course in QL or SumSc.

A1. The course of HRQOL from baseline to 24 months after treatment in relation to variables assessed at baseline


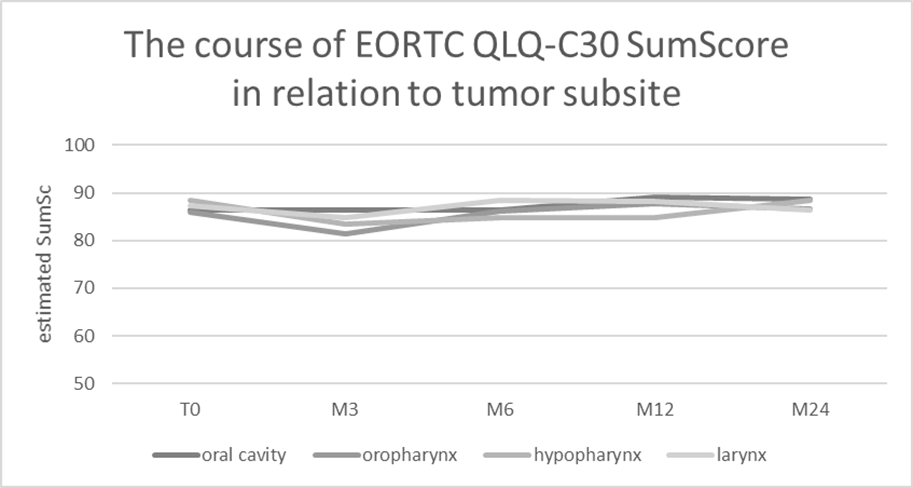


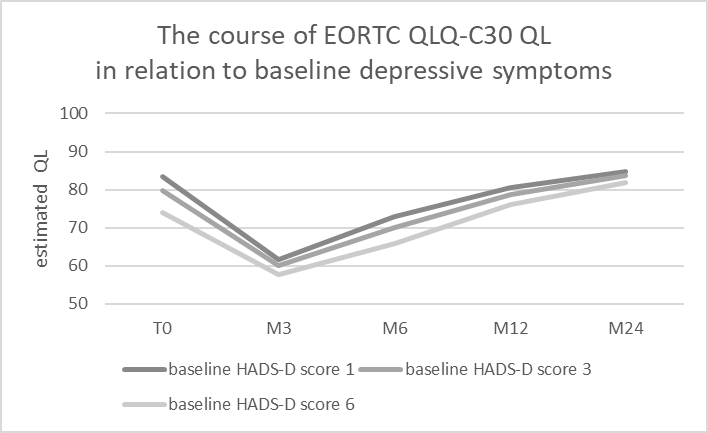


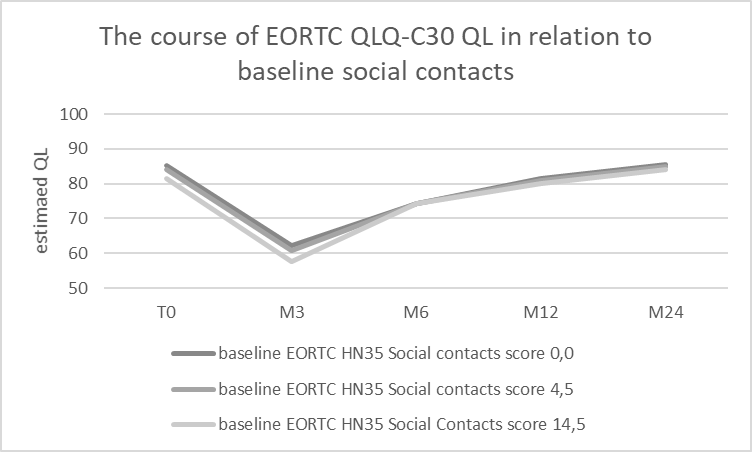


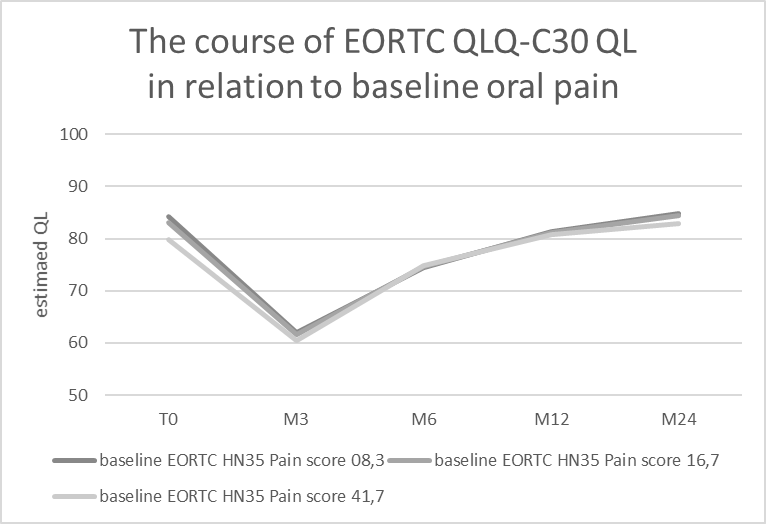


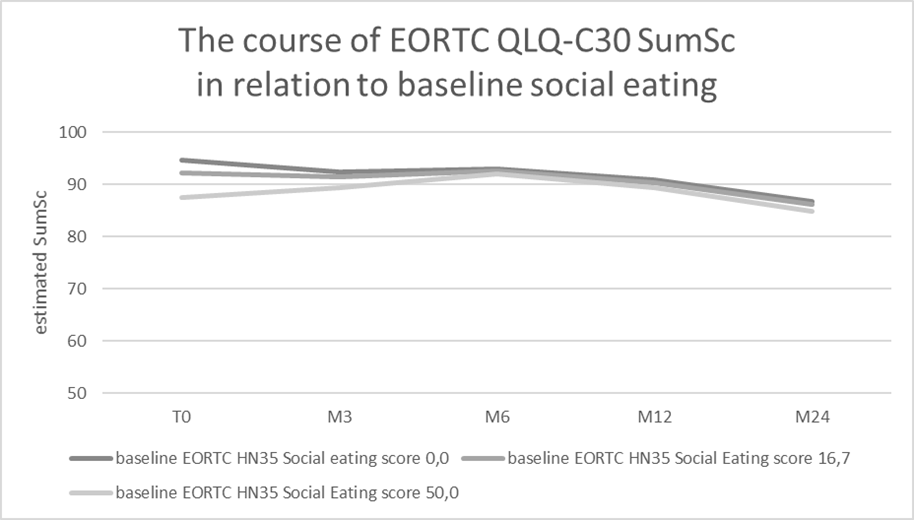


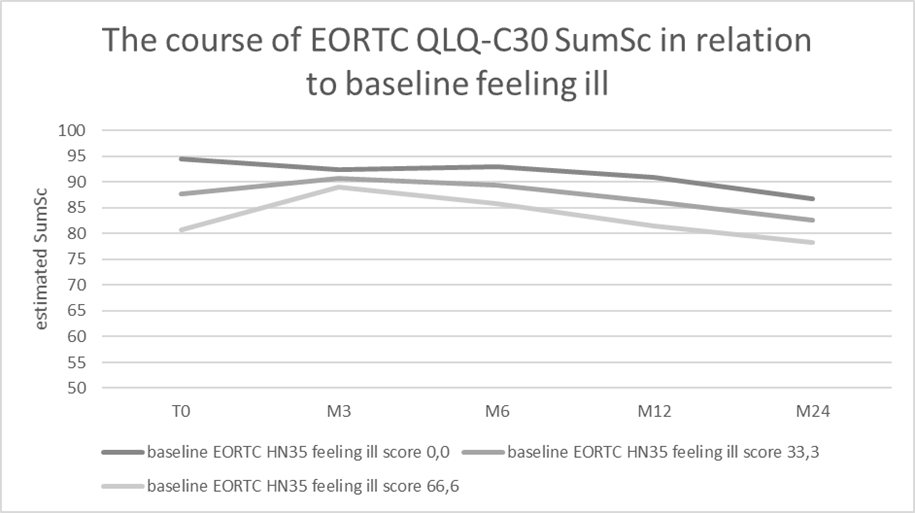


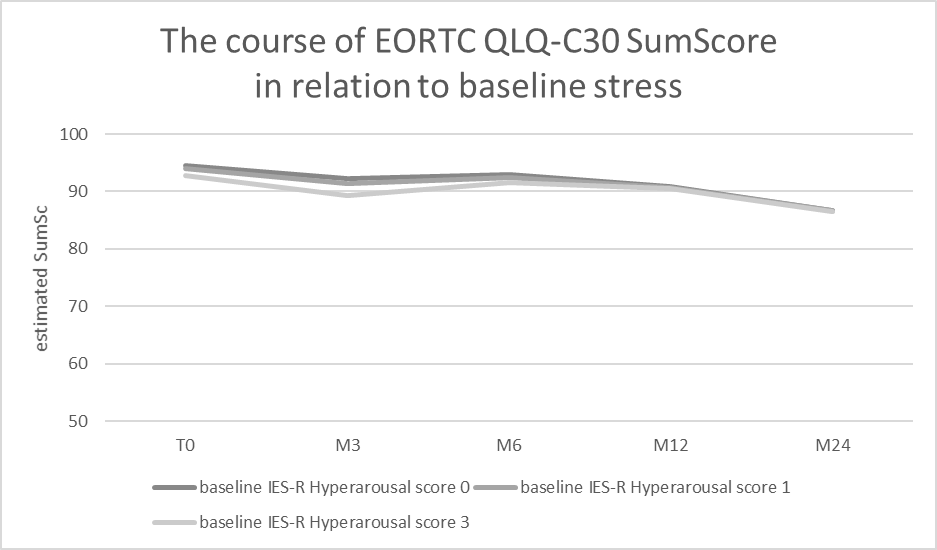


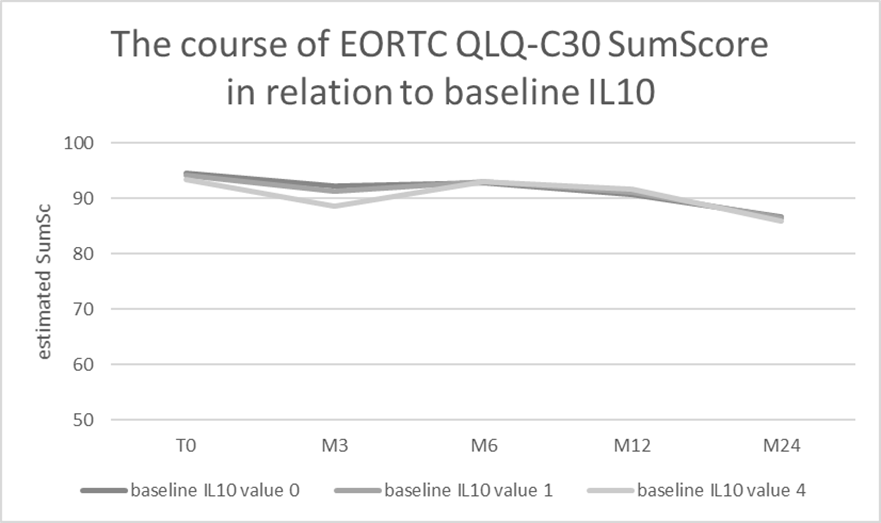


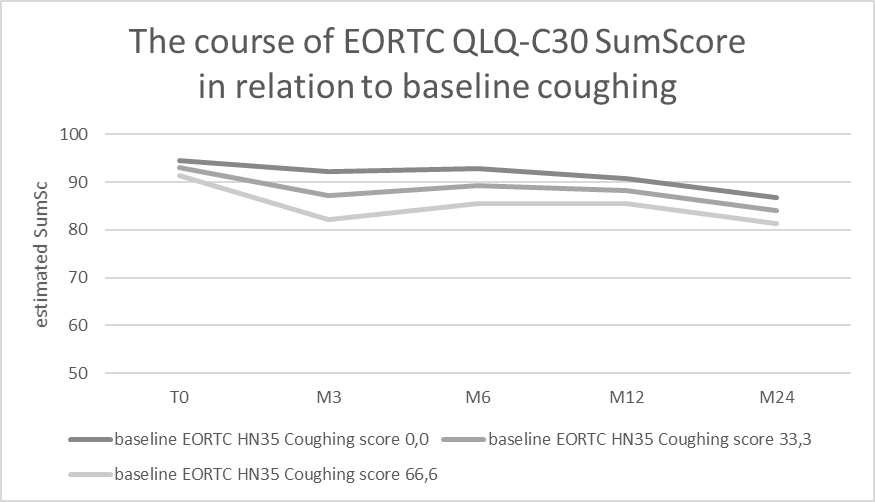


A2. The course of HRQOL from 6 to 24 months after treatment in relation to variables assessed at 6 months after treatment


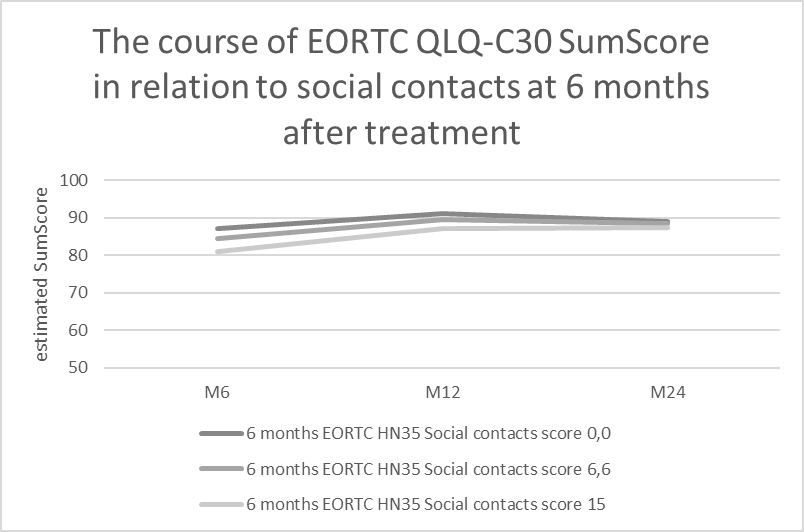


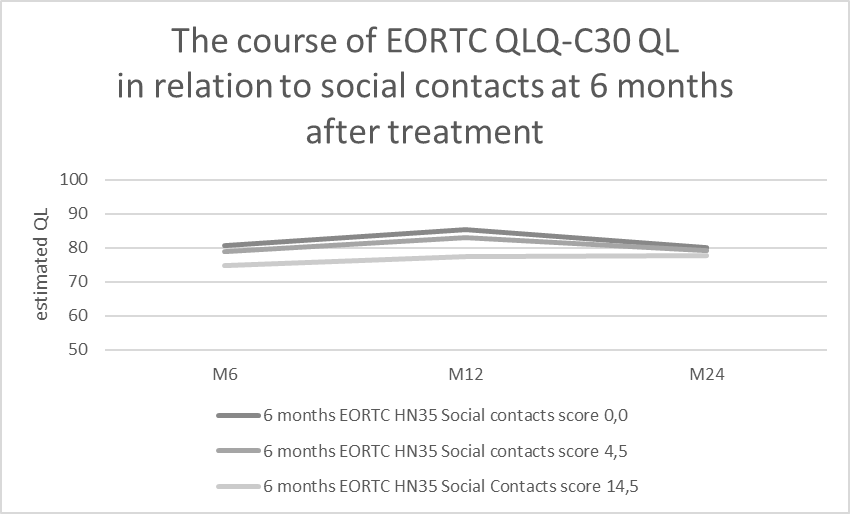


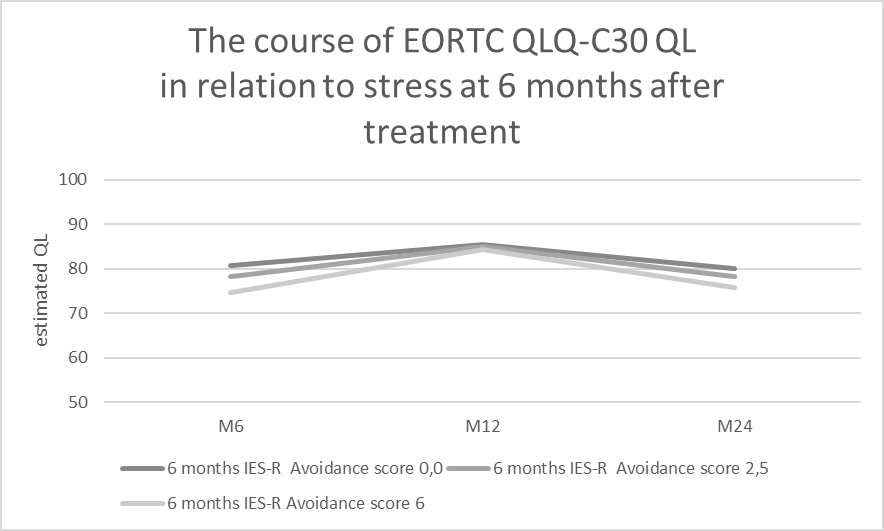


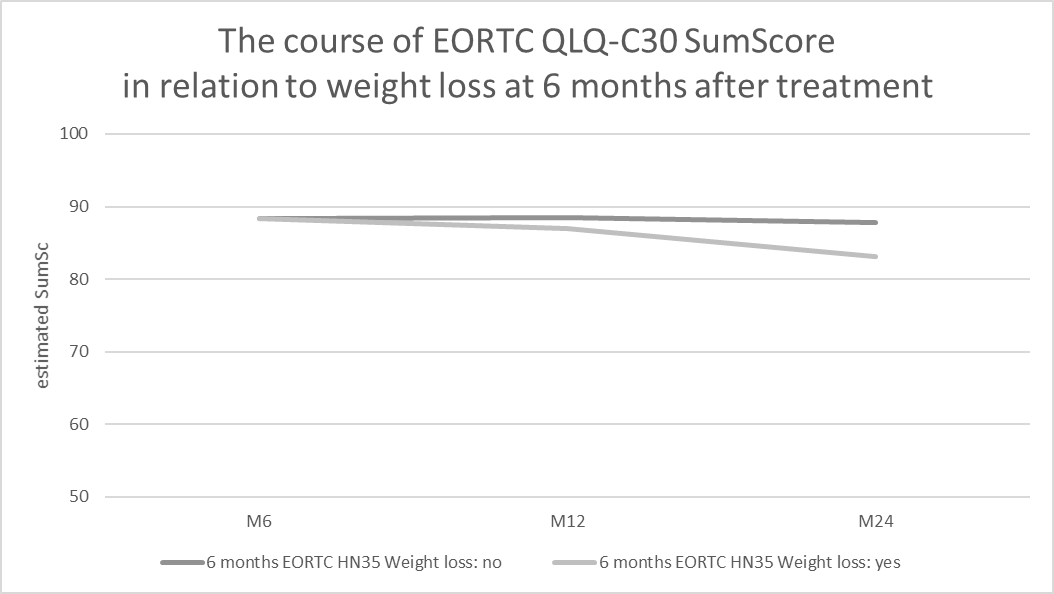


A3. The course of HRQOL from 6 to 24 months after treatment in relation to a change in variables between baseline and at 6 months after treatment


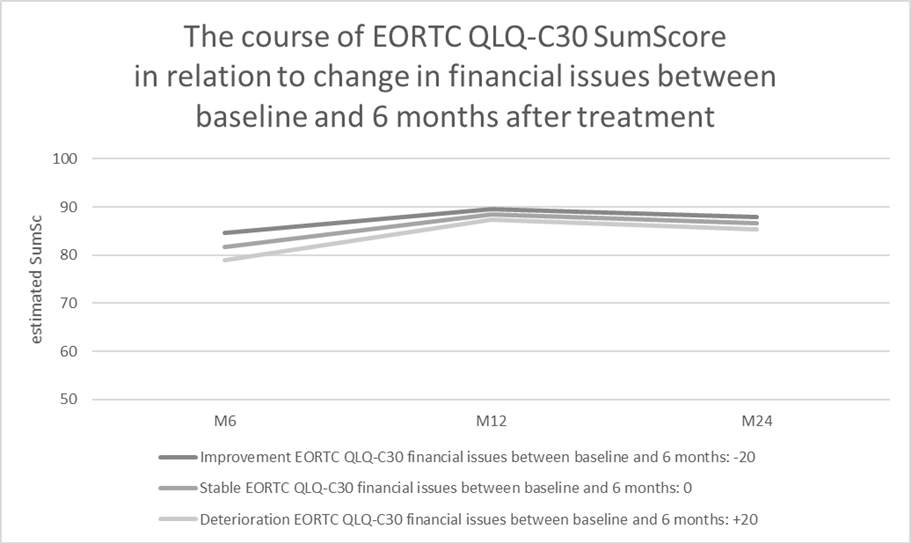


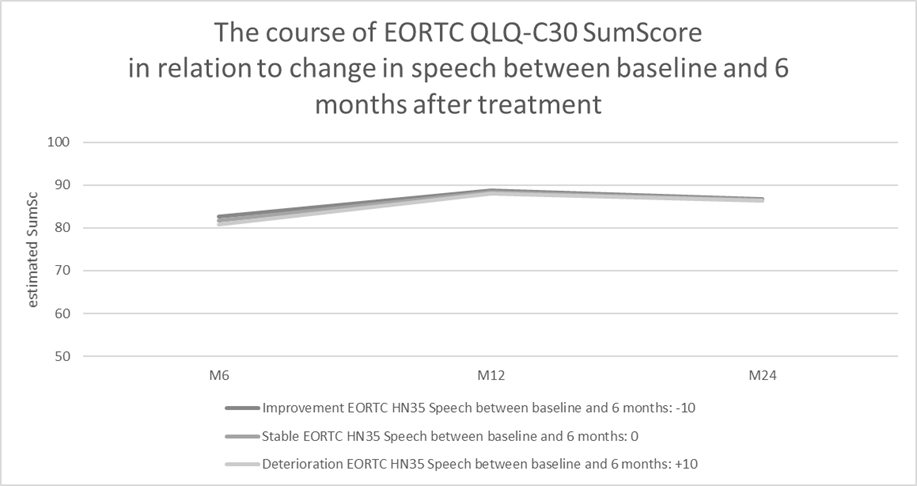


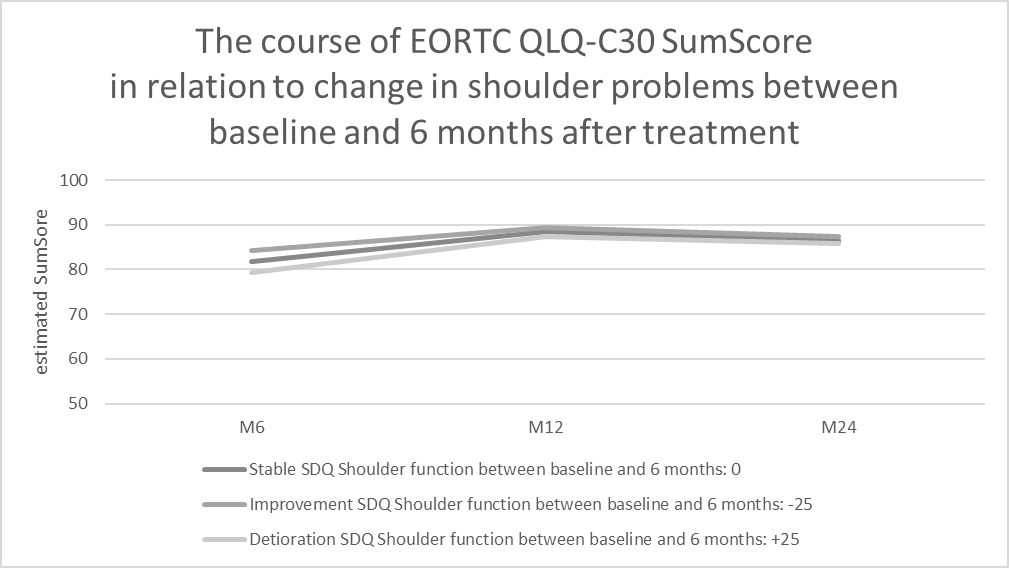


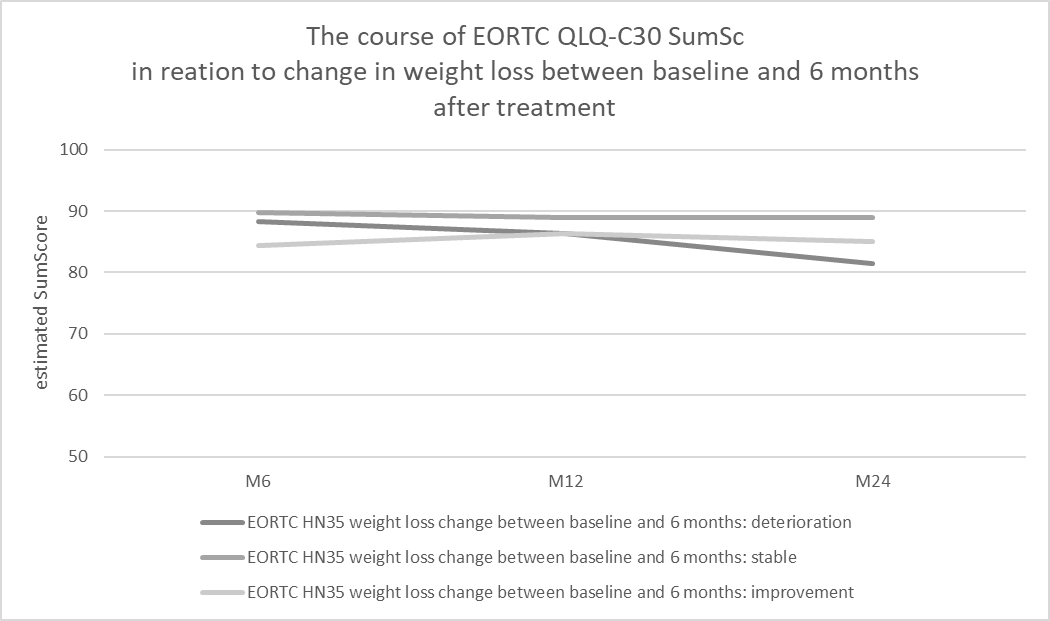


Note: To better visualize the results, the estimates of QL and SumSc are presented on a smaller scale (50-100 instead of 0-100).

Abbreviations:

T0 baseline (after diagnosis, before treatment)); M3, M6, M12, M24L 3, 6, 12, 24 months after treatment; EORTC QLQ-C30 QL/SumSc/HN 35: European Organization for Research and Treatment of Cancer Quality of Life Questionnaire C30 global quality of life scale/sum score/head and neck 35 module; HADS-D: Depression subscale of the Hospital Anxiety and Depression Scale; IES-R Impact of Event Scale-Revised; IL10 Interleukin-10;SDQ Shoulder Disability Questionnaire
